# Loading necrostatin-1 composite bone cement inhibits necroptosis of bone tissue in rabbit

**DOI:** 10.1093/rb/rbz004

**Published:** 2019-02-04

**Authors:** Xiang Ji, Feng Xu, Guoling Dong, Chongzhe Jia, Pu Jia, Hao Chen, Hai Tang

**Affiliations:** Department of Orthopedics, Beijing Friendship Hospital, Capital Medical University, No. 95, Yong’an Road, Xicheng District, Beijing, China

**Keywords:** necrostatin-1, necroptosis, composite bone cement

## Abstract

Bone necrosis after injecting of polymethylmethacrylate (PMMA) bone cement will lead to re-fracture of bone tissue. As a new type of necrosis, there is little research related to the necroptosis of surrounding bone tissue near the bone cement. The purpose of our study was to (i) investigate the presence of necroptosis *in vivo* and, (ii) established as a new type of bone cement containing PMMA, calcium phosphate cement (CPC) and Necrostatin-1 (Nec-1) to inhibit necroptosis of bone tissue. A total of 12 Japanese rabbits were used to establish the animal model and randomly divided into 4 groups signed as a control group, PMMA group, PMMA–CPC group and PMMA–CPC–Nec-1 group, respectively. We used scanning electron microscope to observe the structure of the samples, used HE staining to detect the necrosis, and used western blotting as well as ELISA test to examine the iconic molecule receptor interacting protein kinase-3 (RIP 3) protein and tumor necrosis factor α (TNF-α). After analyzing the results of our study, we found that the structure in both PMMA bone cement group and composite bone cement group was damaged and there was an evidence of necrosis, but it was absent in control group. Through molecule detection, the RIP 3 protein expression was decreased in PMMA–CPC–Nec-1 (*P *<* *0.05). TNF-α expression was increased in bone cement groups with and without CPC (*P *<* *0.05), but was inhibited in PMMA–CPC–Nec-1 group. We have concluded that the necroptosis could be confirmed in bone tissue necrosis induced by TNF-α after bone cement injection and also could be inhibited by composite bone cement with Nec-1.

## Introduction

With the increasing aging of the world, the incidence of osteoporosis and its fractures is increasing year by year [1]. As a common complication of osteoporosis, osteoporotic vertebral compression fracture seriously threatens human health and living standards. Recently, treatments of osteoporotic vertebral compression fracture include nonoperative treatment and surgical treatment, of which percutaneous vertebroplasty (PVP) and percutaneous kyphoplasty (PKP) are the main methods [2]. Some studies have shown that injecting polymethylmethacrylate (PMMA) into the fractured vertebra can significantly relieve pain and improve the quality of patient’s life [3]. However, with the clinical application of this surgery and long-term follow-up after surgery, some studies have found that vertebral re-fracture may occur after PKP and PVP. Moreover, the incidence has been reported in relevant study from 21.6% to 63% [4, 5]. 

As a common bone filling material, PMMA has been used in clinical for a long time due to its advantages, such as injectable property, rapid analgesic effect and good dispersion performance [6]. However, the thermal effect of PMMA bone cement during polymerization and the toxicity of MMA monomer after polymerization may lead to osteonecrosis around bone cement [7, 8]. Recently, Medeiros *et al.* have confirmed by bone tissue sections of vertebral body after PMMA implantation that there is necrosis of cement-bone interface after vertebroplasty and it persists for a certain period of time [9]. The osteonecrosis caused by PMMA implantation resulted in the destruction of bone in the vertebral body and the decrease of bone strength, which eventually led to the occurrence of re-fracture. In order to deal with the disadvantages of PMMA, some studies tried to use new materials to replace PMMA. For example, calcium phosphate cement (CPC), which is an absorbable biomaterial and can be replaced by new bone, was firstly created and used in clinical operation by Brown and Chow in 1985 [10]. Moreover, according to the study of Landerer and Habermacher *et al.* [11], CPC is also used as a carrier for its compatible drugs like antibiotics. But, its biomechanical strength is not as high as PMMA to meet the needs of human body. In the previous studies [10], its degradation rate does not match to the rate of new bone formation and lead to the collapse before forming the new bone.

Necroptosis is a new non-caspase-dependent apoptosis pathway, which is often accompanied by cell dissolution and inflammatory reaction, and has been found in liver, nerve and other tissue damage [12]. Necrostatin-1 (Nec-1) is a specific small-molecule inhibitor of receptor interacting protein kinase-1 (RIP 1) activation in necroptosis pathway which affect expression of its downstream iconic molecule RIP 3 protein at the stage of necroptosis [13, 14]. In other word, if a cell necrosis process can be blocked by Nec-1, the cell death pattern can be confirmed as necroptosis.

There is no relevant study on whether PMMA injection in vertebral compression fracture will lead to necroptosis of bone tissue. In this study, composite bone cement of CPC and PMMA was prepared as a drug carrier to load Nec-1 to investigate the presence of necroptosis *in vivo* and such composite was a modified type of bone cement to inhibit necroptosis of bone tissue.

## Materials and methods

### Preparation of bone cement

In our previous study, *in vitro*, the CPC powder (Ruibang Biological Materials Co., LTD, Shanghai, China) was added to PMMA powder (Heraeus Medical Gmbh, Germany) in different proportions (i.e. 0:1, 1:1, 2:1 and 3:1) and 5.18 mg Nec-1 (MedChemexpress, USA) was added to this mixture. The mass of Nec-1 was calculated according to the Nec-1 reagent specification and referred to other similar experiments [15, 16]. We tested the compression and Nec-1 release level of composite bone cement, and we found that 3:1 of CPC and PMMA have the highest release level but its strength is the lowest, which did not meet the requirement of body strength [17]. Therefore, in this vivo experiment, we chose 1:1 proportion mixture bone cement as the carrier because of its good release performance and biomechanical strength which is similar to the normal physiological state of human vertebrae ∼2–20 Mpa [18, 19].

### Activity detection of released Nec-1 *in vitro*

The 1:1 proportion bone cement sample was chosen to be put into a centrifuge tube with 5 ml PBS in 37°C thermostatic box for releasing Nec-1 for 48 h. After that, the supernatant was collected to co-cultured with MC3T3-E1 cells, which is given by China Infrastructure of cell line resource. The *in vitro* experiment was divided into three groups, including control group, TNF-α group, and TNF-α plus sample releasing supernatant. MC3T3-E1 cells were cultured in 24-well plates at a density of 2 × 10^5^/ml with basal culture media (alpha Modified Eagle Medium, 15% fetal bovine serum, 100 IU penicillin-100μg/ml streptomycin, and 2.5μg/ml Fungizone) for 48 h. Then the medium was changed by fresh medium. After the cells grew to 80% confluence, the collected Nec-1 supernatant was added to co-culture with the cells for 1 h. Subsequently, the necroptosis was introduced by adding 10 μg/ml TNF-α for 48 h culturing. After that, three group cells were treated by trypsinization and then harvested by centrifuging at 3000 rpm for 5 min. Next, according to the manufacturer’s instructions, the cells were suspended and stained with Annexin V and PI by using a FITC Annexin V Apoptosis Kit (MultiSciences Biotech Co., Ltd, China). The experiment was repeated three times independently and the data were analyzed by FlowJo VX software.

### Establishment of animal models

A total of 12 Japanese rabbits were used for the study and randomly divided into 4 groups signed as control group, PMMA group, PMMA–CPC group and PMMA–CPC–Nec-1 group, respectively. At the beginning of the research, we confirmed that every rabbit model was ∼4–6 months old and they weighed between 2000 and 2500 g. Before the experiment, the study was permitted by the Animal Institutional Review Board of the Beijing Friendship Hospital.

At the beginning of the operation, every rabbit was narcotized with ketamine (35 mg/kg) and xylazine (18 mg/kg) via ear vein. Experienced researchers helped us to monitor the operated animal’s vital signs before, during and after the period of anesthetic.

**Figure 1:**
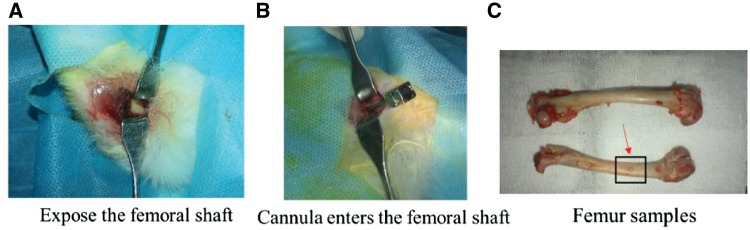
Process of establishing animal models: in Graph C, the red arrow points to the injection point, and black box is the sample intercept rang which centered on the injection point when it is used for protein detection (∼1 cm length and 0.5 cm width)

After anesthesia, the rabbits were placed in supine position, and we shaved their inner skin of both lower limbs. Then the iodophor was used to disinfect the skin. After disinfection, usual sterile fashion was used to draped over the clean skin. A distal femoral incision, ∼0.5 cm, was made at the center of the femur, and then deep muscles were removed to expose some part of the femoral shaft (Fig. 1A). An abrasive drilling was used to drill a hole in the distal end of the femoral shaft, which enabled the trocar used in the vertebroplasty to be fully inserted and ensures that the tip of the cannula enters the femoral shaft (Fig. 1B). We performed a same experiment to determine the amount of bone cement by injecting under maximal pressure, and every femur could be injected between 0.5 and 0.7 ml.

When the trocar is placed well, we removed the central stylet. Then, the bone cement, including PMMA and composite bone cement (CPC:PMMA = 1:1), was prepared in sterile fashion. The powder was mixed with the liquid and then stirred for 1 minute. The CPC and PMMA composite bone cement was prepared as shown previously. A 5-ml syringe was used to extract the mixture and then locked to the trocar. At the distal end of both sides of femur, each animal was injected with 0.5–0.7 ml of cement. The exact amount of cement was recorded in each case. After injecting the cement, we removed the trocar and any excess cement from the operative field subsequently. In the control group, the trocar was placed in the same manner without bone cement injection.

After the operation, normal saline was used to irrigate the incision copiously, and then absorbable sutures were used to close the fascia and skin. The experienced animal researchers monitored their vital signs during their recovery from general anesthesia before they were placed back into their cages. Moreover, they were given some analgesics according to the animal’s physical conditions.

Rabbits were sacrificed by injection of pentothal after the 7 days of surgery and the femur was excised as a whole (Fig. 1C), labeled and subsequently its soft tissue was stripped. And then it was taken an X-ray test (Fig. 4A). The samples were then taken some tests.

### Electron microscope scanning

Every sample was processed in the same manner. Samples were determined from the observed areas and then was fractured mechanically (size: 2 mm×2mm×2 mm). After that, specimens were fixed in 2.5% glutaraldehyde solution in 4°C for 12 h and then they were rinsed by PBS (0.1 M, pH 7.4) for three times, each time for 40 min. The specimens were in turn dehydrated in 50, 70, 80, 90, 95, 100, 100 and 100% ethanol for 15 min each time. Isoamyl acetate was replaced and carbon dioxide was coated with metal spray at the critical point. The bone-cement surface was scanned by a Hitachi-S4800 scanning electron microscope (Hitachi, Japan). The aim of electron microscope scanning is to observe the structural morphology of bone trabeculae.

### Hematoxylin–eosin staining

Histological examination for every femur was taken in the same methods. Firstly, the femurs were fixed in formaldehyde, and then taken ethanol dehydration step by step. After that, they were decalcified in an acid solution. Secondly, the treated samples were stained with hematoxylin and eosin. Thirdly, microtome was used to slice samples. All the procedures were performed according to the guidelines.

An expert pathologist observed the section under microscopy and searched for evidence of bone necrosis, which was defined as there was no osteocytes in its lacunas.

### Western blotting analysis

The femur was excised as a whole unit and then its soft tissue was stripped. Next, the femur was sliced up 0.5 cm above and below the center of the injection point along the axial direction of femur shaft (shown in Fig. 1C), and then had ground at low temperature with a grinder (Tissuelyser-32, Jingxin company, Shanghai). After that, the powder was collected and dissolved in radioimmunoprecipitation assay cleavage buffer containing 4% protease inhibitors, 4% phosphorylase inhibitors, 4% PMSF for 1 h at low temperature. After centrifugation (5000 rpm, 30 min), the supernatants were aspirated and their protein concentrations were determined. An aliquot of each sample was separated on a 10% sodium dodecylsulfate-polyacrylamide gel and transferred onto a nitrocellulose membrane (Hybond ECL, Amersham Biosciences, Freiburg, Germany). The membranes were incubated in blocking buffer (5% nonfat milk powder in TBST [TBS with 0.1% Tween 20]) for 2 h and then incubated overnight at 4°C with gentle shaking with specific primary antibody directed against RIK3 (diluted 1:500). After the membrane was washed, it was incubated at room temperature for 2 h with a secondary antibody (ZSGB-BIO of China). The membranes were visualized with enhanced chemiluminescence (GE Healthcare, Piscataway, NJ, USA).

### ELISA test

The femurs were processed and ground as the same method as described above. Then the powder was mixed with PBS (weight volume ratio: 1:9) and added with phosphatase inhibitors. After repeated shaking, the homogenate was centrifuged at 5000 rpm for 30 min, and the supernatant was collected, and then tested according to the operation instructions of the kit (Mlbio, Shanghai).

### Statistical analysis

All statistical analyses were performed with SPSS 22.0 software (SPSS, Chicago, IL, USA) and GraphPad Prism6 software. Means ± standard deviations was used to express the data, and the unpaired Student’s t-test was performed to determine the differences among groups. Significance was determined at a *P* values of <0.05.

## Results

### Flow cytometric analysis *in vitro*

**Figure 2:**
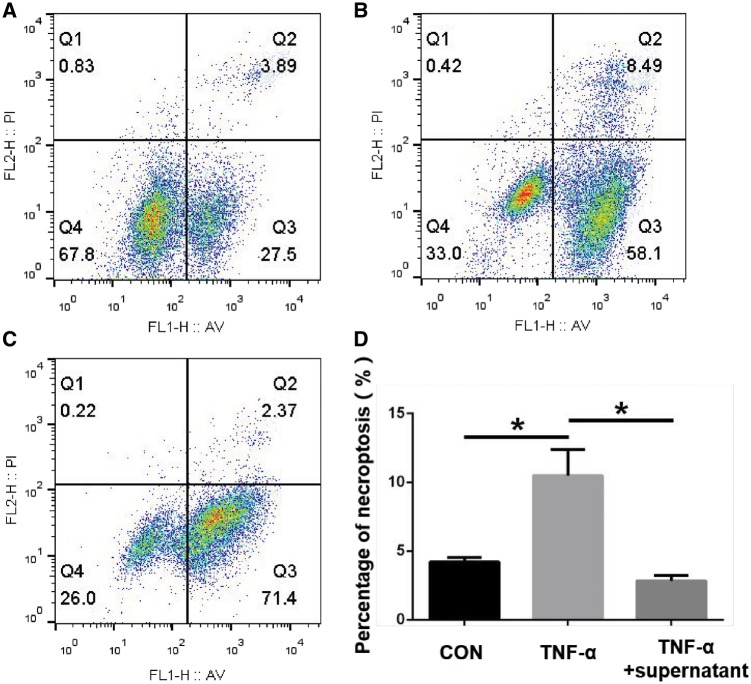
Flow cytometric analysis of MC3T3-E1 cell *in vitro*: (**A**) control group; (**B**) TNF-α group; (**C**) TNF-α plus sample releasing supernatant group; (**D**) analysis of necroptosis cells percentage.(* represents that there is significant difference between two groups, *P *<* *0.05)

TNF-α can induce cell necroptosis as shown in other experiment, and the Nec-1 is a specific inhibitor of the pathway [20]. According to that, the cell experiment was to detect the activity of released Nec-1. In Fig. 2, compared with control group, the percentage of necroptosis cells in TNF-α group increased significantly from 3.89 to 8.49%. But in TNF-α plus sample releasing supernatant group, it decreased obviously to 2.37%. Three independent experiments were repeated and the mean percentage of necroptosis in the 3 groups were (4.22 ± 0.18)% in control group; (10.49 ± 1.09)% in TNF-α group and (2.83 ± 0.23)% in TNF-α plus sample releasing supernatant group, respectively (*n* = 3, *P *=* *0.0048 between control group and TNF-α group, *P *=* *0.0024 between TNF-α group and TNF-α+ releasing supernatant group, Fig. 2).

### Results of scanning electron microscopy

**Figure 3:**
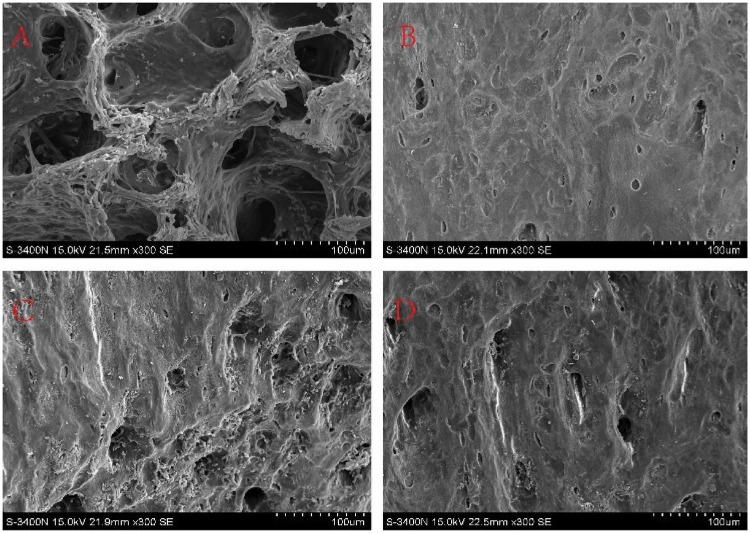
Electron microscopy scanning diagram of samples (magnified by 300 times): (**A**) control group; (**B**) PMMA; (**C**) PMMA+CPC; (**D**) CPC+PMMA+NEC-1

As seen in Fig. 3, Graph A is control group in which the trabecular bone structure is clear and complete with obvious bone lacunae and visible cells. Graph B in comparison to the Graph A, the trabecular structure of the PMMA group (as CPC:PMMA = 0:1 group) was damaged, bone lacunae were not clear and a small amount of PMMA material was left.

The trabecular structure was damaged and the bone lacunae was also not clear in the Graph C and D (CPC + PMMA group and CPC + PMMA + Nec-1 group, the ratio of CPC and PMMA =1:1) in compared with Graph A but they were better than Graph B. Moreover, there were some bone cells in the bone lacunae and some composite bone cement material was left. While Comparing Graph C with D, there was no significant difference between these two groups.

### X-ray image and HE staining results

**Figure 4:**
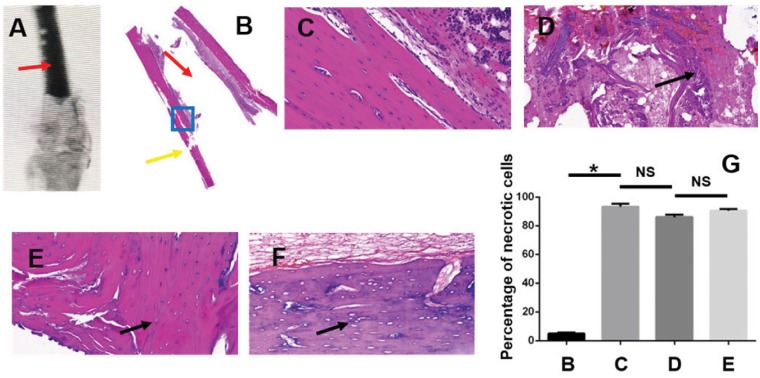
X-ray image and HE staining of the femurs(magnified by 20 times): (**A**) X-ray of the sample implanted bone cement (the red arrow indicates bone cement material injected into the bone tissue); (**B**) overall view of the HE staining sample (the blank part indicated by the red arrow is filled with PMMA material, the yellow arrow indicated the injection point, and the blue box is the observation area); (**C**) control group; (**D**) PMMA group: the black arrow shows the necrotic tissue; (**E**) PMMA+CPC group; (**F**) CPC+PMMA+NEC-1 group. In Graphs E and F, the black arrow shows the osteocytes were lost in the lacuna; (**G**) the comparison of percentage of necrotic cells between four groups (showed in C, D, E, F. * represents that there is significant difference between two groups, *P *<* *0.05; NS shorts for there is no significant difference between two groups, *P *>* *0.05)

The Graph A showed the X-ray image of the sample which was injected with bone cement, and in this picture, the red arrow indicates the bone cement material. HE staining was performed to test whether there was bone necrosis or not when bone cement was injected into bone tissue. Graph B in Fig. 4 was the overall view of the HE stained sample, in which the blank part indicated by the red arrow is filled with PMMA material, the yellow arrow indicated the injection point and the blue box is the observation area. Graphs C, D, E and F were the four groups pictures, respectively. Graph C was control group, which showed that the bone cortex and medullary cavity were clear, as the cell distribution in the bone lacuna is clearly visible. In Graph D (PMMA group), the structure of bone was destroyed and unclear, and necrotic debris appeared (as indicated by the arrow). Graphs E and F were PMMA + CPC group and PMMA + CPC + Nec-1 group, respectively, in which the trabecular was also destructed, and the osteocytes were lost in the lacuna. Graph G compared the percentage of necrotic cells between four groups, and it demonstrated that after filling bone cement, the necrotic cells percentage increased dramatically (*P *<* *0.05) and there was no significant difference between D, E and F (*P *>* *0.05).

### Expression of proteins associated with necroptosis

**Figure 5:**
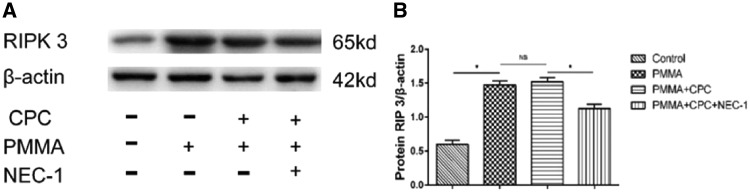
The expression of RIP 3 protein by western blotting (* represents that there is significant difference between two groups, *P *<* *0.05; NS shorts for there is no significant difference between two groups, *P *>* *0.05)

**Figure 6:**
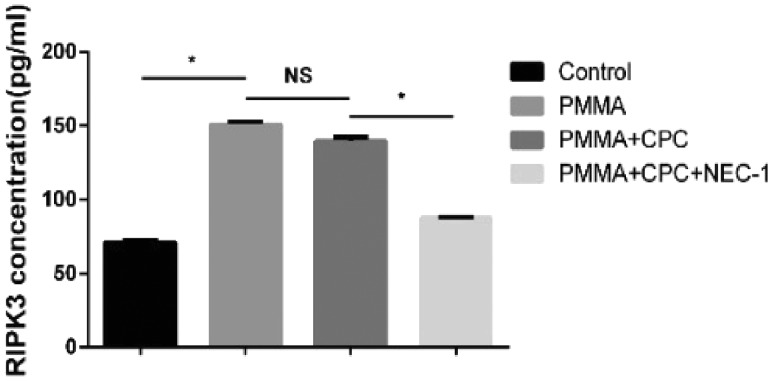
The expression of RIP 3 protein by ELISA test (* represents that there is significant difference between two groups, *P *<* *0.05; NS shorts for there is no significant difference between two groups, *P *>* *0.05)

A previous study shows that RIP 1 plays an important role in necroptosis, which can mediate the necroptosis [13, 14]. When RIP 1 was inhibited by Nec-1 (a specific inhibitor of RIP 1 kinase activity), the complex of RIP 1 and RIP 3 will be inhibited. Through western blotting and ELISA detection, we investigated whether RIP 3 expression will be affected after injection of bone cement with Nec-1. The expression of RIP 3 protein was examined at 7 days after the operation. As shown in Fig. 5A and B, RIP 3 protein expression was significantly upregulated in PMMA group and composite bone cement group, and as Nec-1 was added into the composite bone cement, the protein level was reduced clearly. These data demonstrate that the Nec-1 inhibits RIP 1 activation, resulting in the expression and aggregation of the downstream RIP 3. Then, we use ELISA assay as an accurate detection to detect the protein concentration quantitatively, which result (Fig. 6) was consistent with the trend of western blotting test.

### Result of TNF-α expression

**Figure 7:**
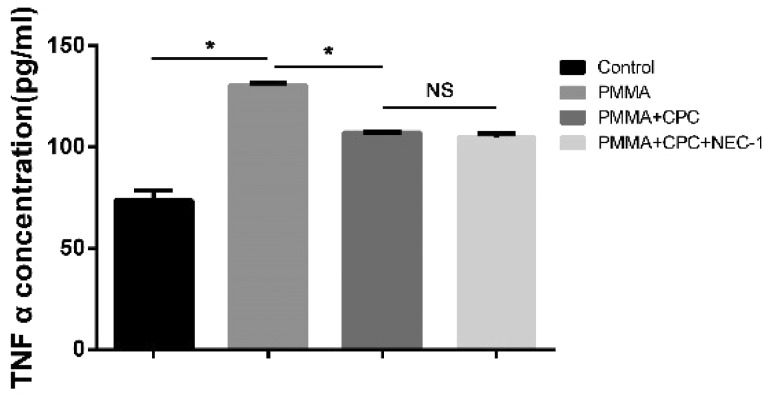
The ELISA test result of TNF-α protein expression (* represents that there is significant difference between two groups, *P *<* *0.05; NS shorts for there is no significant difference between two groups, *P *>* *0.05)

TNF-α was considered as the mean reason of bone necrosis, and it has been confirmed to induce cell necroptosis [20–22]. In this study, through ELISA test of the TNF-α concentration, shown in Fig. 7, after injection of bone cements or composite bone cements, the concentration in Groups B, C and D were significantly increased than the group A (*P *<* *0.05). Compared with Group B, Group C was decreased significantly. But there was no significant difference between groups C and D (*P *>* *0.05).

## Discussion

PVP and PKP are commonly used in vertebral compression fracture which is a common disease among elderly patients and seriously affects the quality and safety of life [1, 2]. In PVP or PKP operations, PMMA bone cement is the most commonly used bone filling materials in clinical practice due to its some obvious advantages. However, according to some researches [23], the disadvantages of PMMA such as thermal effect, nonabsorbability, excessive biomechanical strength and poor loading performance, can also result in vertebrae-surrounding tissue necrosis and refracture of the fractured vertebrae. *In vitro* study [17], the release level is always at a low level in PMMA. Some researches have suggested that in antibiotic-loaded PMMA bone cement experiments, antibiotics were released mainly in the first few hours and then released slowly over time [23]. In order to reduce the complications caused by PMMA, CPC which is a kind of absorbable and drug-loadable bone cement [24], is applied to replace PMMA. But it also has some defects such as excessive degradation and low biomechanical strength which limit its application. In our previous study *in vitro* [17], a composite bone cement was prepared by mixing a certain proportion of CPC and PMMA which takes advantage of CPC to compensate for the disadvantages of PMMA and improve the drug-carrying performance of PMMA materials. And in this study, we also tested the activity of the releasing Nec-1 *in vitro*, which showed that it could inhibit the necroptosis pathway when culturing with MC3T3-E1 cells treated by TNF-α.

In this study, HE staining result indicated that there was obvious bone necrosis in PMMA group which was also reported in previous study [8]. In composite bone cement groups, namely PMMA–CPC group and PMMA–CPC–Nec1 group, the bone necrosis was not as evident as PMMA group, but also can be observed due to the absence of osteocytes in lacunas. By electron microscopy scanning, the trabecular structure was damaged in three groups except control group. As the addition of CPC, the destruction of trabecular structure was not as serious as PMMA group. These results suggested that injection of bone cement including composite bone cement could lead to the bone structural failure and bone tissue necrosis. We consider that mechanical damage and thermal effects of PMMA during bone cement injection are the causes of bone necrosis around cement which is considered as the reasons of affecting the bone tissue recovery and leading to *in situ* collapse.

Necroptosis is a new pathway of cell death, also called programmed necrosis. It is also mediated by RIP 1 protein, involved in all cell death pathways, and its downstream RIP 3 protein, which only involved in necroptosis pathway [25]. Nec-1 is the specific inhibitor of necroptosis. In other word, if a cell death pathway is inhibited by Nec-1 then the pathway is necroptosis pathway. In this study, western blotting and ELISA test had shown that the RIP 3 protein expression was increased in bone cement groups including PMMA group, PMMA–CPC group and PMMA–CPC–Nec-1 group compared with the control group (*P *<* *0.05). This has demonstrated that injection of bone cement could result in bone necrosis which is the same result as HE staining and electron microscopy scanning. Compared with PMMA group and PMMA–CPC group, we found that the RIP 3 protein expression had clearly decreased in PMMA–CPC–Nec-1 group (*P *<* *0.05) which means that the necrosis pathway was inhibited by Nec-1. Thus, it suggests that the necrosis caused by bone cement includes the necroptosis pathway and loading Nec-1 composite bone cement could reduce the occurrence of necroptosis after injecting bone cement which may play a role in decreasing the risk of re-fracture.

Recently, some studies had shown that TNF-α plays an important role in necroptosis which could induce the necroptosis pathway [20–22]. When bone cement injects into the fracture vertebrae, bone cement could lead to the inflammation of bone tissue in which TNF-α protein is one of the important products. TNF-α could induce RIP 1 protein interact with tumor necrosis factor receptor type 1-associated death domain and then collect RIP 3 protein to initiate the necroptosis pathway progress. In this study, the TNF-α expression was significantly increased among three bone cement groups through ELISA test (*P *<* *0.05). Therefore, injection of bone cement leads to increase TNF-α expression, and then TNF-α induces bone necrosis. By loading Nec-1, we found that RIP 3 protein expression was decreased, which means the pathway was inhibited by Nec-1.

In summary, our study is to confirm the existence of necroptosis in the bone tissue necrosis caused by bone cement, and we can use the composite bone cement loading Nec-1 to protect bone cell against necroptosis.

## Conclusion

The composite bone cement of PMMA and CPC with a certain proportion can achieve the biomechanical strength to meet physiological implantation requirement. At the same time, 1:1 mixed bone cement is a stable drug-loading system and release drug effectively. In addition, the necroptosis is confirmed in bone tissue necrosis induced by TNF-α after bone cement injection and could be inhibited effectively through composite bone cement with Nec-1.

### Limitation

One limitation of our study is that we verify the existence of necroptosis after injecting bone cement to bone tissue, however, the necroptosis is one but not the only one pathway of cell death, and there should be multiple necrosis pathway. In order to reduce the refracture caused by bone necrosis after injection of PMMA, other necrosis pathway should be researched and blocked in the future studies, which could be better to reduce the risk of refracture.

## Funding

This study was supported by the National Nature Science Foundation of China (NNSFC No. 81472086). 

*Conflict of interest statement*. None declared.
